# Function and Molecular Ecology Significance of Two Catechol-Degrading Gene Clusters in *Pseudomonas putida* ND6

**DOI:** 10.4014/jmb.2009.09026

**Published:** 2020-12-11

**Authors:** Sanyuan Shi, Liu Yang, Chen Yang, Shanshan Li, Hong Zhao, Lu Ren, Xiaokang Wang, Fuping Lu, Ying Li, Huabing Zhao

**Affiliations:** 1 Tianjin Key Laboratory of Industrial Microbiology, College of Biotechnology, Tianjin University of Science and Technology, Tianjin 300457, P.R. China; 2 Department of Environmental Science and Engineering, Xi'an Jiaotong University, Xi'an 710048, P.R. China; 3 Tianjin Entry-Exit Inspection and Quarantine Bureau, Tianjin 00457, P.R. China; 4 China Animal Disease Control Center, Beijing 100000, P.R. China

**Keywords:** *Pseudomonas putida* ND6, catechol *ortho*-cleavage pathway, CatR_I_ and CatR_II_, *catB_I_C_I_A_I_* and *catB_II_C_II_A_II_* operons, cross-regulation, evolution of catabolic pathways

## Abstract

Many bacteria metabolize aromatic compounds via catechol as a catabolic intermediate, and possess multiple genes or clusters encoding catechol-cleavage enzymes. The presence of multiple isozyme-encoding genes is a widespread phenomenon that seems to give the carrying strains a selective advantage in the natural environment over those with only a single copy. In the naphthalene-degrading strain *Pseudomonas putida* ND6, catechol can be converted into intermediates of the tricarbo*xyl*ic acid cycle via either the *ortho*- or *meta*-cleavage pathways. In this study, we demonstrated that the catechol *ortho*-cleavage pathway genes (*catB_I_C_I_A_I_* and *catB_II_C_II_A_II_*) on the chromosome play an important role. The *cat_I_* and *cat_II_* operons are co-transcribed, whereas *catA_I_* and *catA_II_* are under independent transcriptional regulation. We examined the binding of regulatory proteins to promoters. In the presence of *cis*-*cis*-muconate, a well-studied inducer of the cat gene cluster, CatR_I_ and CatR_II_ occupy an additional downstream site, designated as the activation binding site. Notably, CatR_I_ binds to both the *cat_I_* and *cat_II_* promoters with high affinity, while CatR_II_ binds weakly. This is likely caused by a T to G mutation in the G/T-N_11_-A motif. Specifically, we found that CatR_I_ and CatR_II_ regulate *catB_I_C_I_A_I_* and *catB_II_C_II_A_II_* in a cooperative manner, which provides new insights into naphthalene degradation.

## Introduction

A number of aerobic biodegradation pathways of aromatic compounds like benzoate, aniline, phenol, pyrene and naphthalene converge at the catechol ring cleavage reaction, and eventually generate intermediates of the tricarbo*xyl*ic acid cycle through two different routes [1-3]. The catechol *meta*-cleavage pathway is usually encoded by the *nah* and *xyl* genes on plasmids, while the catechol *ortho*-cleavage pathway is usually determined by the chromosomal cat and ben genes [4-9]. *Ortho*-cleavage is followed by further steps of the β-ketoadipate pathway which is catalyzed by the three enzymes catechol 1,2-dioxygenase (C12O, E.C. 1.13.11.1) (*catA*), *cis*,*cis*-muconate-lactonizing enzyme I (*catB*), and muconolactone isomerase (*catC*). The expression of this operon is regulated by CatR, and is induced by *cis*,*cis*-muconate (CCM) [9-15].

Several research groups have found that bacteria possess multiple catechol *ortho*-cleavage genes or clusters, which might indicate the importance of keeping the intracellular concentration of catechol and its derivatives low [6,7, 16-20]. Notably, the strains with multiple catechol dioxygenase genes can usually grow on many more aromatic compounds than strains with one dioxygenase gene [14, 21]. Different enzymatic properties and induction patterns of C12O isozymes may be responsible for the metabolism of different substrates [22]. For example, three different C12O isozymes encoded by *catA*, *catA_I_* and *catA_II_* in the naphthalene-degrading strain *Pseudomonas putida* ND6 were found to have different enzymatic properties [17]. *Burkholderia* sp. strain TH2, a 2-chlorobenzoate (2CB)-degrading bacterium, metabolizes benzoate (BA) and 2CB via two catechol *ortho*-cleavage pathways (*cat_1_* and *cat_2_*). Interestingly, the inducer of *catA_1_* was found to be BA, and not 2CB. It was also found that CCM or its metabolite acts as an inducer for *catA_2_*. These results suggest that although *cat_2_* genes are not indispensable for growth of TH2 on 2CB, they are advantageous [20]. Murakami *et al*. reported that the aniline-assimilating bacterium *Frateuria* sp. ANA-18 has two *cat* clusters, which are respectively located on the chromosome and the large plasmid [19]. Two CatR proteins that differ in their binding affinity for the *catB* promoters control the expression of the two cat gene clusters, which is affected by the concentration of CCM. The *catB_1_* promoter was shown to be active in the presence of CCM, while the *catB_2_* promoter was activated only at low concentrations of CCM [23]. Hence, the presence of multiple catechol *ortho*-cleavage genes and pathways in the same cell seems to be a widespread phenomenon that gives the host strains a selective advantage in the natural environment. However, the research on function, regulation and evolutionary significance of these genes and clusters should be further intensified.

*P. putida* strain ND6, which was isolated by our group from industrial wastewater in Tianjin, China, was capable of growth on naphthalene as the sole carbon and energy source, and successfully degraded 98% of 2 g/l naphthalene in mineral medium in 48 h. Similar to other described strains [24-26], ND6 possesses an integrated naphthalene-degrading pathway, and catabolizes catechol through the *meta*-cleavage pathway. However, a unique feature of ND6 exists in the presence of two catechol *ortho*-cleavage pathways, encoded by *catR_I_B_I_C_I_A_I_* and *catR_II_B_II_C_II_A_II_* on the chromosome, in addition to the typical catechol *meta*-cleavage pathway on the plasmid. We investigated the cellular responses of *P. putida* ND6 grown in medium with 2 (normal concentration) and 4 g/l naphthalene (high concentration) using a quantitative proteomics-based approach. Comparative analysis of the proteomic data indicated that the expression levels of CatA_I_, CatB_I_, and CatB_II_, which are involved in the catechol *ortho*-cleavage pathway, were upregulated [27]. This demonstrated that *P. putida* ND6 is able to survive in the presence of high naphthalene concentrations mainly because of the duplicated catechol *ortho*-cleavage operons. The two *ortho*-cleavage operons are regulated by the LysR-type regulatory proteins CatR_I_ and CatR_II_, respectively. The distance between the two clusters was about 754 kb, and their sequence identity was 73.57%. Based on the previous work in our laboratory, we hypothesized that these two gene clusters have different functions and evolutionary origins, and might regulate each other via genetic cross-talk. In this paper, we compared the function, regulation and evolutionary significance of the two catechol *ortho*-cleavage gene clusters in *P. putida* strain ND6.

## Materials and Methods

### Bacterial Strains, Culture Conditions, Plasmids, Primers and DNA Manipulations

*P. putida* strain ND6 and mutants were cultured at 30°C and 180 rpm in Luria-Bertani (LB), or in mineral medium (MMB) with 0.2% naphthalene or 0.2% glucose as the carbon source. *Escherichia coli* strains were grown in LB at 37°C and 180 rpm. Where appropriate, spectinomycin (Sp; 50 mg/l), streptomycin (Sm; 50 mg/l), chloramphenicol (Cm; 25 mg/l), ampicillin (Amp; 50 mg/l), kanamycin (Kan; 50 mg/l), carbenicillin (Cb; 75 mg/l), tetracycline (Tet; 10 mg/l), or gentamicin (Gm; 10 mg/l) was added for selection.

The bacterial strains, plasmids, and primers used in the present study are listed in Tables 1 and 2. All DNA manipulations were performed according to standard procedures [31]. Restriction enzymes, DNA polymerase and T4 DNA ligase were used in accordance with the manufacturers’ specifications.

### Construction of *P. putida* ND6 Mutants

The suicide vector pEX18Tc was used for gene replacement in *Pseudomonas*. The *catR_I_* replacement vector pEX18Tc-R_1_Km was constructed by inserting the Kan^r^ cassette into the *catR_I_* gene in the pEX18Tc vector to generate a partial deletion from 226 to 440 bp. Similarly, the *catR_II_* replacement vector pEX18Tc-R_II_Km was constructed by replacing 335 to 407 bp, and the *catA* replacement vector pEX18Tc-AKm by replacing between 10 to 514 bp. The knockout plasmids pEX18Tc-RIKm, pEX18Tc-R_II_Km, and pEX18Tc-AKm were transferred from *E. coli* S17-1 into *P. putida* ND6 by intergeneric conjugation , followed by selection on LB + Cb + Kan medium with 20% sucrose to generate the *catR_I_*, *catR_II_* and *catA* mutants. The resulting mutant strains were named *P. putida* ND6-Δ*catR_I_*, ND6-Δ*catR_II_*, and ND6-Δ*catA*, respectively.

The *nahH* replacement vector pEX18Tc-HCm was constructed by inserting the Cm^r^ cassette into the internally deleted *nahH* gene in the pEX18Tc vector, replacing the sequence between 204 to 742 bp. Finally, the *P. putida* strain ND6-Δ*catAnahH* was obtained.

### Time-Courses of Growth, Naphthalene Degradation, and Catechol Dioxygenase Activity

The wild-type ND6, as well as the mutants ND6-Δ*catR_I_*, ND6-Δ*catR_II_* and ND6-Δ*catAnahH*, were individually grown in MMB medium with 0.2% naphthalene. Bacterial growth was monitored by measuring the optical density at 600 nm. Naphthalene in the culture broth was extracted twice with an equal volume of dichloromethane, and its concentration was measured according to a published method [32]. The activity of catechol dioxygenases (C12O and C23O) was assayed as described previously [17].

### Quantitative Real-Time PCR (RT-qPCR)

The wild-type ND6, as well as the ND6-Δ*catR_I_* and ND6-Δ*catR_II_* mutants were cultured at 30°C in MMB with glucose or naphthalene. Cells were harvested at 25 h (end of the exponential growth phase) and washed with TE buffer. RNA was extracted using the RNAprep Pure Bacteria Kit (Tiangen Biotech, China) and reverse-transcribed into cDNA using the FastKing RT Kit (with gDNase) (Tiangen Biotech). The RT-qPCR was performed following the instructions of the Green Premix Ex Taq II Kit. All RT-qPCR reactions were conducted in three biological and three technical replicates.

Plasmid standards were used for absolute quantification of the corresponding gene fragments. The plasmid copy number was determined according to the molar mass derived from the plasmid and amplicon sequences [33]. For each standard sample, the RT-qPCR system was used to measure the cycle threshold values, which were used to draw a standard curve for each isoform by plotting the cycle threshold values versus the log value of the transcript copy number. Regression equations generated by the system software were used to calculate the transcript copy number for each isoform in each test sample, which was normalized to the value of the 16S rRNA and expressed as the absolute copy number per 1000 copies of 16S transcript. The 95% confidence interval was used to assess the significance of copy number differences, and analysis of variance (ANOVA) was used to assess the significance of the threshold cycle values.

### Expression and Purification of CatR_I_ and CatR_II_

*E. coli* BL21 carrying the CatR_I_ and CatR_II_ expression plasmids pEX5-CatR_I_ and pEX5-CatR_II_ were grown overnight in LB medium with kanamycin. The expression was induced overnight at 16°C by adding a final concentration of 0.5 mM isopropyl-β-D-thiogalactopyranoside (IPTG). To purify CatR_I_ and CatR_II_, cell pellets obtained from 1 liter cultures were collected at 13,000 ×*g* for 3 min at 4°C and resuspended in 40 ml of binding buffer (50 mM sodium phosphate, 0.3 M NaCl, pH 7.4), and sonicated. The clarified lysate was applied to a nickel-nitrilotriacetic acid (NTA) affinity column and allowed to bind for 1 h, followed by washing with binding buffer containing 10 mM, 30 mM and 60 mM imidazole, and eluting with binding buffer containing 500 mM imidazole. The collected fractions were determined by sodium dodecyl sulfate-polyacrylamide gel electrophoresis (SDS-PAGE). The desalination column Sephadex G-25 (GE, USA) was used to remove the imidazole, and yielded the purified protein. The protein was stored in glycerol at -20°C.

### Primer-Extension Assay

The primer extension assay was carried out according to the protocol of Fekete *et al*., as published previously [34].

### Electrophoretic Mobility Shift Assay (EMSA)

The fluorescent 6-fluorescein amidite (FAM)-labeled probes were prepared by amplifying the promoter regions of pUC19c-catB_I_ and pUC19c-catB_II_ by PCR using the primers M13F-11 and M13R-12 (Table 2). The Wizard SV Gel and PCR Clean-Up System (Promega) was used to purify the resulting FAM-labeled probes, which were then quantified using a NanoDrop 2000C instrument (Thermo Fisher Scientifc, USA). The EMSA samples comprised 20 μl of 50 mM Tris-HCl [pH 8.0], 100 mM KCl, 2.5 mM MgCl2, 0.2 mM dithiothreitol (DTT), with 2 μg salmon sperm DNA and 10% glycerol, as well as 40 ng of the probe and the indicated DNA-binding proteins. After incubation for 30 min at 30°C, the mixture was loaded onto a 10% PAGE gel buffered with 0.5×Tris-borate-EDTA (TBE).

### DNase I Footprinting

The fluorescent FAM-labeled probes were prepared as described above. The DNase I footprinting assays were conducted as published by Wang *et al*. [35]. For each assay, 400 ng of the probe in a total volume of 40 μl was mixed with different amounts of the indicated DNA-binding protein. The preparation of the DNA ladder, electrophoresis and data analysis were the same as described before [35], except that the GeneScan-LIZ500 size standard (Applied Biosystems) was used.

### Construction of *catB_I_*-*lacZ* and *catB_II_*-*lacZ* Fusions and β-Galactosidase Activity Assays

A 309 bp DNA fragment corresponding to the *catR_I_*-*catB_I_* intergenic region (from -347 to -39 relative to the *catB_I_* initiation codon) and a 330bp DNA fragment corresponding to the *catR_II_*-*catB_II_* intergenic region (from -358 to -29 relative to the *catB_II_* initiation codon) were amplified by PCR to construct *cat_I_* promoter-*lacZ* fusion and *cat_II_* promoter-*lacZ* fusion, respectively. Two amplified fragments of *cat_I_* and *cat_II_* promoter were digested with EcoRI and BamHI, respectively, and inserted into pDN19lacΩ that had been previously cut with the same enzymes. The resulting plasmids, pDB_I_ and pDB_II_, were transformed into *E. coli* S17-1 by chemical transformation. The complementation was performed through the conjugation between *E. coli* S17-1 with plasmid pDB_I_ and pDB_II_ and *P. putida* ND6, *P. putida* ND6-Δ*catR_I_*, and ND6-Δ*catR_II_*. Recombinant *P. putida* was selected on MMB plate containing 0.2% salicylate (w/v %) with Cb, Sp and Sm, and subcultured several times to ensure plasmid stabilization. The β-galactosidase activity was measured according to the method of Green [31].

### Phylogenetic Tree and Analysis of Conserved Sequences

Database searches were performed using BLAST at the National Center for Biotechnology Information (NCBI) website. Multiple sequence alignments were performed using Clustal W. Then, a neighbor-joining (NJ) phylogenetic tree was constructed using MEGA software version 5.0, and the branching reliability was tested using bootstrap re-sampling (1,000 pseudo-replicates). Analysis of conserved motifs based on known sequences was conducted using WebLogo to generate LOGO diagrams.

## Results

### Functional Analysis of the Two Catechol-Degrading Gene Clusters

**Fig. 1:**
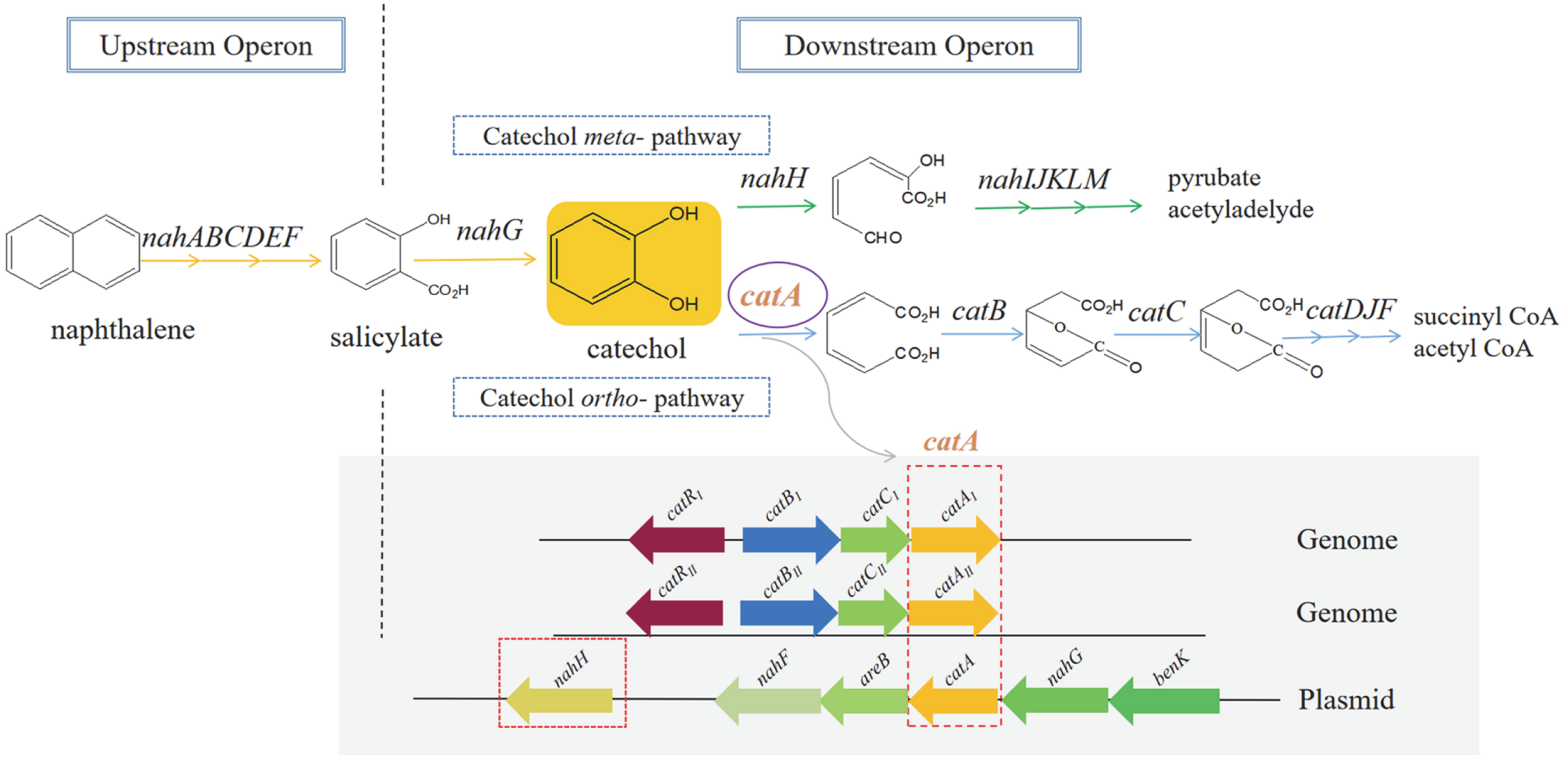
Genetic, regulatory and biochemical connections of naphthalene catabolism in *P. putida* ND6. The figure sketches the pathways involved in the naphthalene catabolism of *P. putida* ND6: the upstream operon (*nah*ABCDEF) for the conversion of naphthalene into salicylate; the downstream operon for the conversion of salicylate into catechol and eventually into intermediates of the central carbon metabolism. Catechol metabolism can proceed via either the *ortho*- or the *meta*-cleavage pathway. Both pathways converge towards catechol and diverge at that point, as this compound can be cleaved between positions 1 and 2 by *catA*, whereas *nahH* cleaves between positions 2 and 3. The three *catA* genes are located on the chromosome and large plasmid, respectively.

**Fig. 2:**
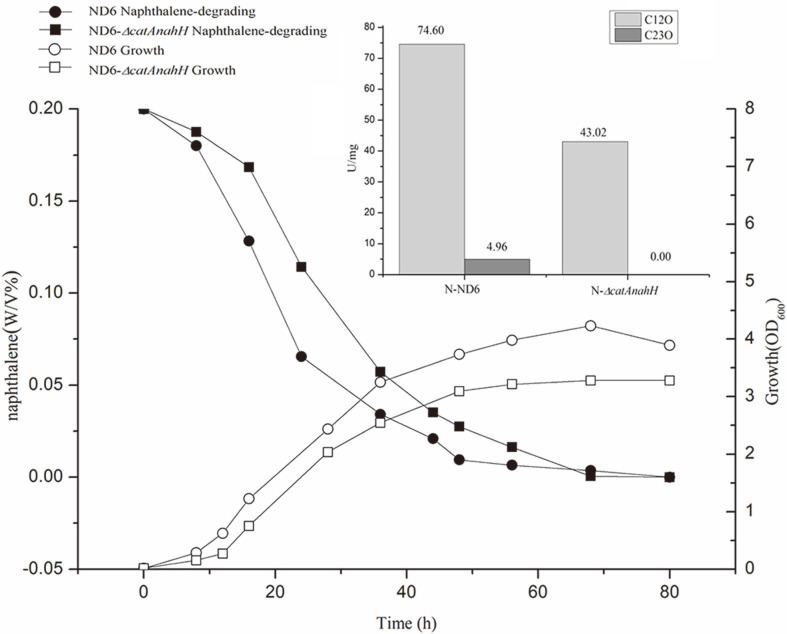
Naphthalene-degradation phenotype and catechol dioxygenase enzyme activities of *P. putida* ND6 and the ND6-Δ*catAnahH* mutant. Naphthalene (0.2%, w/v %) was provided as the sole carbon source. The small figure shows the C12O and C23O enzyme activity of *P. putida* ND6 and ND6-Δ*catAnahH*.

**Fig. 3:**
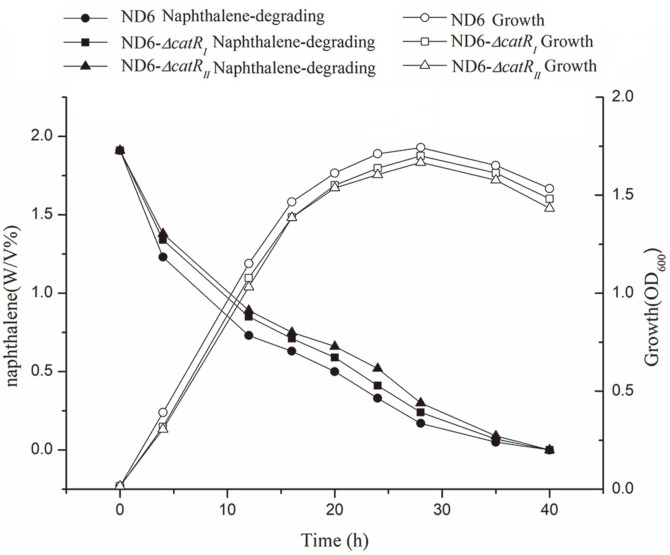
Growth and naphthalene-degradation curves of *P. putida* ND6 wild type, as well as the mutants ND6-Δ*catR_I_* and ND6-Δ*catR_II_*. Naphthalene (0.2%, w/v) was provided as the sole carbon source.

As mentioned above, the genome of *P. putida* ND6 encodes two catechol-degrading gene clusters (*catR_I_B_I_C_I_A_I_* and *catR_II_B_II_C_II_A_II_*) (Fig. 1). To investigate the possible physiological role of these two orthologous clusters, *catA* (encoding C12O) and *nahH* (encoding C23O) from the large plasmid pND6-1, which are responsible for catechol ring cleavage [16], were knocked out. The obtained double-mutant strain ND6-Δ*catAnahH* was still able to grow on naphthalene by employing the catechol-degrading gene clusters in the genome as a backup, although the C12O activity dropped from 74.60 to 43.02 U/mg (Fig. 2). Furthermore, to examine the relative roles of the *cat_I_* and *cat_II_* clusters in catechol metabolism, we disrupted the *catR_I_* and *catR_II_* regulatory genes and examined the properties of the resulting strains. The ability of these strains to use naphthalene as the sole carbon source was tested in growth and degradation experiments. Interestingly, inactivation of *catR_I_* or *catR_II_* had no obvious effect on the strains’ ability to utilize naphthalene (Fig. 3).

### Transcriptional Analysis of the Two Catechol-Degrading Gene Clusters

**Fig. 4:**
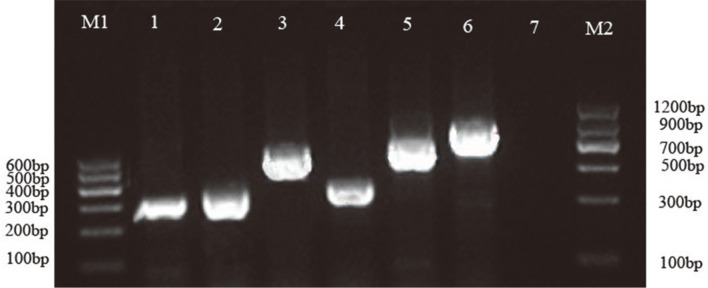
Agarose gel plots were used to determine the transcription patterns of the two cat gene clusters. M1: Tiangen DNA Marker I; M2: Tiangen DNA Marker II; Lane1: Band of the *catB_I_*-*catC_I_* intergenic region DNA fragment; Lane 2: Band of the *catC_I_*-*catA_I_* intergenic region DNA fragment; Lane 3: Band of the *catB_I_*-*catA_I_* intergenic region DNA fragment; Lane 4: Band of the *catB_II_*-*catC_II_* intergenic region DNA fragment; Lane 5: Band of the *catC_II_*-*catA_II_* intergenic region DNA fragment; Lane 6: Band of the *catB_II_*-*catA_II_* intergenic region DNA fragment; Lane 7: negative control.

**Fig. 5:**
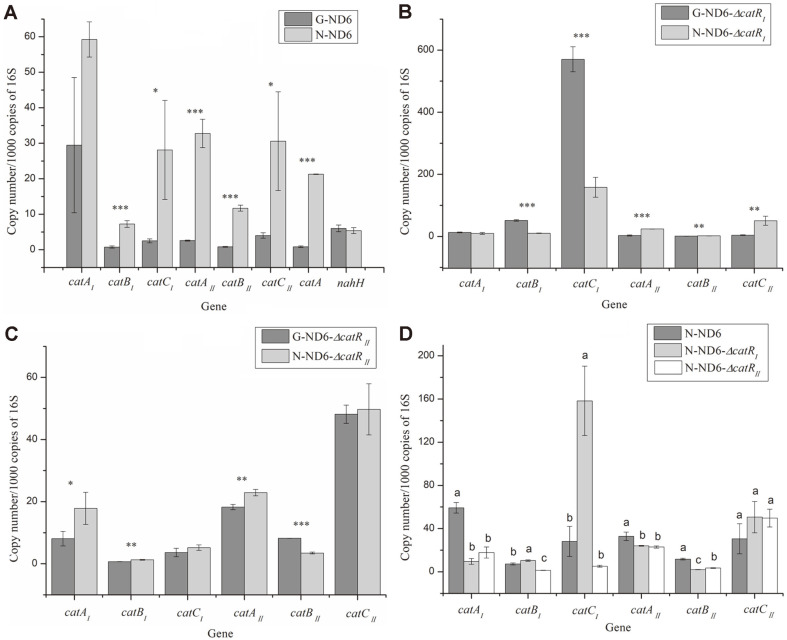
Copy number determination by absolute RT-qPCT using plasmid standards. Transcript copy number of genes per 1000 mRNA transcript copies of the 16S rRNA in ND6 (**A**), ND6-Δ*catR_I_* (**B**), and ND6-Δ*catR_II_* (**C**). *p < 0.05, **p < 0.01, ***p < 0.001 between cells grown on naphthalene and on glucose. (**D**) Transcript copy number of two cat gene clusters per 1000 mRNA transcript copies of the 16S rRNA in ND6, ND6-Δ*catR_I_* and ND6-Δ*catR_II_*. Different superscript letters indicate statistically significant differences among experimental groups (*p* < 0.05; Duncan’s multiple range test). Either naphthalene (N-) or glucose (G-) was provided as the sole carbon source. Bars show the standard errors of the means.

The cDNA of *P. putida* ND6 was used as template to amplify the intergenic regions of the *catB_I_C_I_A_I_* and *catB_II_C_II_A_II_* operons. The results indicated that either the *catB_I_C_I_A_I_* or *catB_II_C_II_A_II_* cluster can be co-transcribed (Fig. 4). However, the results of RT-qPCR revealed that the transcription of the downstream genes *catC_I_* and *catA_I_* was higher than that of *catB_I_* (Fig. 5A), suggesting that *catC_I_* and *catA_I_* were independently transcribed, while the *catB_I_C_I_A_I_* cluster was co-transcribed. A similar phenomenon was also observed in the cluster *catB_II_C_II_A_II_* (Fig. 5A).

In the naphthalene medium, the activity of transcription (Fig. 5A) and expression levels (Fig. 2, small figure) of three *catA* genes and C12O were much higher than *nahH* gene and C23O. Therefore, the naphthalene metabolism of *P. putida* ND6 proceeds via catechol cleavage in both the *ortho* and *meta* positions, whereby the *ortho*-cleavage pathway is the main cleavage pathway. The transcriptional levels of the *ortho*-cleavage pathways (*catA_I_*, *catB_I_* *catC_I_*, *catA_II_*, *catB_II_*, *catC_II_*, *catA*) were all increased in naphthalene medium, while that of *meta*-cleavage pathways (*nahH*) was not (Fig. 5A). It indicates that the *ortho*-cleavage pathways can be induced by naphthalene or its metabolites but the *meta*-cleavage pathway apparently cannot. Furthermore, we measured the transcriptional levels of *cat_I_* and *cat_II_* in the mutant strains ND6-Δ*catR_I_* and ND6-Δ*catR_II_*, following cultivation in the presence of glucose or naphthalene. For the ND6-Δ*catR_I_* strain (only *catR_II_* is functional), the transcriptional levels of *catB_I_* were significantly decreased, while the *catB_II_* gene cluster could still be induced (Fig. 5B). For the ND6-Δ*catR_II_* strain (only *catR_I_* is functional), the transcriptional level of *catB_II_* was also decreased while the *catB_I_* gene cluster could still be induced (Fig. 5C). This result demonstrated that CatR_I_ and CatR_II_ are the native regulatory proteins of the *cat_I_* cluster and the *cat_II_* cluster, respectively.

To investigate their possible regulatory roles, the impacts of *catR_I_* and *catR_II_* were further analyzed by determining the transcriptional levels of all six catechol-degradation genes in wild-type ND6 and the mutants when grown with naphthalene as the sole carbon source. When either *catR_I_* or *catR_II_* was knocked out, the transcription of the *cat_I_* or *cat_II_* cluster could still be detected (Fig. 5D). It indicates that CatR_I_ and CatR_II_ might cross-regulate the transcription of each other's target genes, and explains that the naphthalene degradation curves of ND6-Δ*catR_I_* and ND6-Δ*catR_II_* did not exhibit significant changes (Fig. 3).

### Promoter Structure of the Two Catechol-Degrading Gene Clusters

**Fig. 6:**
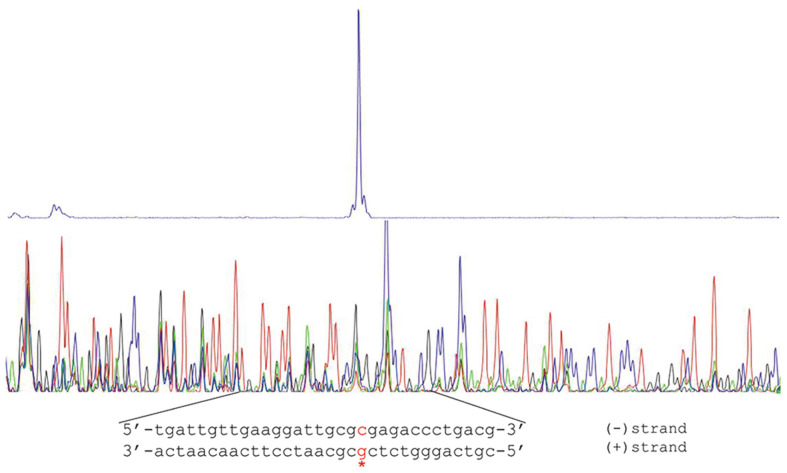
Results of the primer-extension assay. Characterization of the *cat_1_* transcription start site (TTS) using the primer extension assay. The primer extension experiments used the primer *catB_I_*-pe (Table 2), which was designed to bind downstream of the *catB_I_* initiation codon. Comparison of the sequencing results of the cDNA (above) and the genomic DNA (below) to determine the TSS (marked with a red asterisk).

The primer-extension assay indicated that the transcription start site (TSS) of *cat_I_* is located at a G nucleotide (Fig. 6). Surprisingly, the primer extension assay of *cat_II_* failed three times based on reliable methods, probably because the transcript abundance of the *cat_II_* cluster is particularly low.

**Fig. 7:**
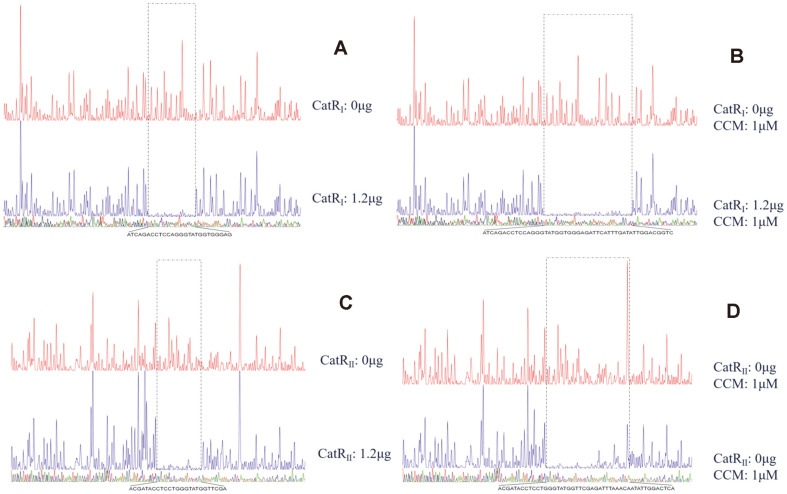
DNase I footprinting analysis of CatR_I_ (A, B) binding to the *catB_I_* promoter and CatR_II_ (C, D) binding to the *catB_II_* promoter with or without CCM. Based on these results and earlier reports on bacterial promoter prediction [39], we identified the promoter structure of the two catechol-degrading gene clusters (Fig. 8). The putative TSS of *cat_I_* was consistent with the primer-extension experiment, and the putative TSS of *cat_II_* was located 67 bp upstream of the *catB_II_* initiation codon. Other motifs of the *cat_I_* and *cat_II_* promoters were identified by comparing to the reported conserved sequences recognized by RNA polymerase [11, 13, 19, 23, 40, 41], including the –35 element (positions –35 to –30, if the transcriptional start site is denoted as +1), the –10 element (positions –12 to –7) and the discriminator element (Dis; –6 to –4) (Fig. 8).

**Fig. 8:**
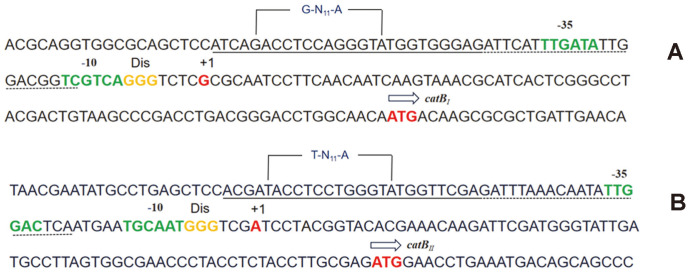
Structure of the *catB_I_* and *catB_II_* promoter regions. (**A**) The promoter region of the *cat_I_* gene cluster. (**B**) The promoter region of the *cat_II_* gene cluster. The transcription start site is shown as +1. The predicted discriminator element (Dis), initiation codon (arrows, the direction represents the direction of transcription), and -10 and -35 regions of the promoter were also designated above the sequence. The promoter regions of *cat_I_* and *cat_II_* protected by CatR_I_ or CatR_II_ from DNase I digestion without CCM, called the repression binding site (RBS), are marked with black solid lines; the extended protected region in the presence of CCM, called the activation binding site (ABS), is marked with the black dotted line.

The CatR_I_ and CatR_II_ binding regions were also studied using DNase I footprinting (Fig. 7). Purified CatR_I_ was incubated with a 408 bp fragment labeled with FAM, with a sequence corresponding to the promoter region of *cat_I_*, and the resulting complexes were treated with DNase I, followed by DNA fragment analysis by capillary electrophoresis. The binding sequence of the CatR_II_-*catB_II_* complex was assessed using the same method, and a 403 bp FAM-labeled DNA fragment was incubated with purified CatR_II_. In the absence of CCM, CatR_I_ protected a continuous 26 bp region that had also been determined to be located from -137 to -112 relative to the *catB_I_* codon (Fig. 7A). CatR_II_ protected a continuous 25 bp region that had also been determined to be located from -137 to -113 relative to the *catB_I_* initiation codon (Fig. 7C). In the presence of CCM, CatR_I_ protected a continuous 48 bp region that was determined to be located from -137 to -90 relative to the *catB_I_* initiation codon (Fig. 7B). CatR_II_ protected a continuous 41 bp region that was determined to be located from -137 to -97 relative to the *catB_I_* initiation codon (Fig. 7D). These results demonstrated that CCM has a significant effect on the DNA binding mode of CatR. In the absence of CCM, CatR bound the repression binding site (RBS), which might be associated with the negative regulation of itself. In the presence of CCM, CatR occupied an adjacent downstream site (Fig. 8), designated as the activation binding site (ABS) [36, 37]. Like most LysR proteins, the DNA-binding sites of CatR_I_ and CatR_II_ invariably contain incomplete inverted repeats (G-N_11_-A) or inverted repeats (T-N_11_-A), respectively [38]. The results also indicated that the -35 and -10 regions of the cat promoter were important for promoter activity but not for CatR binding.

### Transcriptional Cross-Regulation between the Two Catechol-Degrading Gene Clusters

**Fig. 9:**
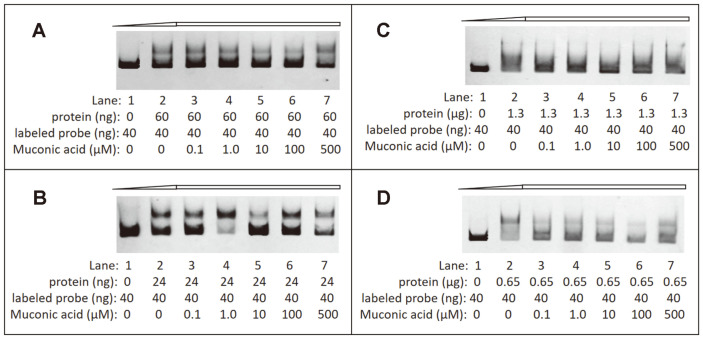
EMSA of CatR_I_ and CatR_II_ binding to the *cat_I_* and *cat_II_* promoters, respectively. (**A**) EMSA of CatR_I_ binding to the *cat_I_* promoter. (**B**) EMSA of CatR_II_ binding to the *cat_II_* promoter. (**C**) EMSA of CatR_I_ binding to the *cat_II_* promoter. (**D**) EMSA of CatR_II_ binding to the *cat_I_* promoter. The first lane contains the free fragment, lanes 2-7 contain labeled probe and CatR_I_ or CatR_II_ protein, with 0, 0.1, 1.0, 10, 100, and 500 μM muconic acid, respectively. The concentration of the labeled probe was fixed at 40 ng.

The regulatory proteins CatR_I_ and CatR_II_ were cloned into a vector to introduce six histidine residues at the C-terminus of the protein, and were expressed and purified from *E. coli* as described in the Materials and Methods section. The binding abilities of CatR_I_ and CatR_II_ to the upstream regulatory regions of *cat_I_* and *cat_II_* were studied by EMSA using a concentration gradient of CCM (Fig. 9). As expected, the addition of 60 ng CatR_I_ to 40 ng of the labeled probe caused a strong shift in the mobility of the *cat_I_* promoter fragment (Fig. 9A). Notably, CatR_I_ could also strongly bind to the *cat_II_* promoter region (Fig. 9C). By contrast, CatR_II_ was able to specifically bind the *cat_II_* and *cat_I_* promoter regions, but the binding stability of the CatR_II_-*cat_II_* and CatR_II_-*cat_I_* complexes was obviously reduced (Figs. 9B and 9D). These results support the hypothesis that CatR_I_ and CatR_I_ might cross-regulate the transcription of each other's target genes. In addition, CCM did not affect the binding of CatR_I_ to the corresponding target regions, which was in agreement with previous reports [10, 13]. However, the binding regions in the presence and absence of CCM might be different. As can be seen in Figs. 9B anddding CCM to the reaction mixture decreased the intensity of the DNA band, indicating that CatR_II_ recognizes and binds to the *cat_II_* and *cat_I_* promoters when CCM is at a lower concentration.

### Promoter Activity Analysis of the Two Catechol-Degrading Gene Clusters

The primer extension assay indicated that there might be significant differences in the abundance of transcripts between the *cat_I_* and *cat_II_* clusters. To test this idea, the promoter activities of *cat_I_* and *cat_II_* (transcriptionally fused to the *lacZ* gene in pDN19lacΩ) were examined using the classical reporter β-galactosidase (Table 3).

As expected, in strain NP2 (ND6 wild type containing the *cat_II_* promoter-*lacZ* fusion), the β-galactosidase activity was low in both glucose and naphthalene media. By contrast, the β-galactosidase activity of strain NP1 (ND6 wild type containing the *cat_I_* promoter-*lacZ* fusion) was very high, indicating that the *cat_I_* promoter is much stronger than the *cat_II_* promoter. The activity of the *cat_II_* promoter in cells grown on glucose or naphthalene showed little variation, probably because the transcript abundance was too low. The β-galactosidase activity of NP1 cells grown on glucose was approximately 75.9% of the activity of cells grown on naphthalene, indicating that naphthalene or its metabolites can slightly induce the *cat_I_* promoter but are not necessary for its activity.

To confirm the function of CatR_I_, we introduced the plasmid pDB_I_ (pDN19lacΩ + *cat_I_* promoter) and plasmid pDB_II_ (pDN19lacΩ+*cat_II_* promoter) into ND6-Δ*catR_I_* and ND6-Δ*catR_II_*, respectively, resulting in the strains ND1P1 and ND1P2. A drastic reduction of β-galactosidase activity was observed in ND1P1, whereby the promoter activity of *cat_I_* was almost equal to that of the control strain NC (ND6 containing pDN19*lac*Ω) in glucose-grown cells, but the *cat_I_* promoter could be still induced slightly in naphthalene-grown cells. This result suggested CatR_II_ might function as a backup regulator. Interestingly, the promoter activity of *cat_II_* was also decreased in the CatR_I_ mutant (ND1P2) compared with NP2, suggesting that CatR_I_ might competitively bind the promoter region of *cat_II_* and initiate transcription more effectively in the wild-type ND6. On the other hand, we found that the β-galactosidase activity of ND2P1 (ND6-Δ*catR_II_* containing pDN19lacΩ + *cat_I_* promoter) was obviously higher than that of NP1, both on glucose and on naphthalene. This result suggested that CatR_I_ could bind the *cat_II_* promoter and initiate transcription more efficiently with or without the inducer when CatR_II_ is unavailable. All these results were in agreement with the EMSA results. Through in vitro and in vivo experiments, we have confirmed that two regulatory proteins CatR_I_ and CatR_II_ have interactive regulation on the *cat_I_* and *cat_II_* gene clusters, and CatR_I_ has a stronger regulatory and activation effect on the gene clusters than CatR_II_.

### Evolutionary Analysis of the Two Catechol-Degrading Gene Clusters and Their Promoter Regions

**Fig. 10:**
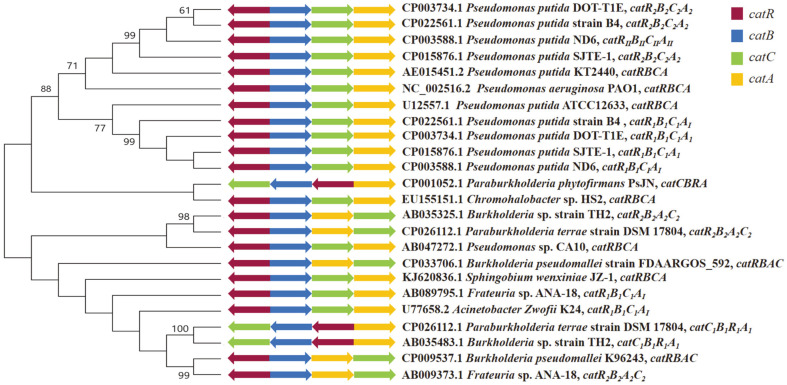
Phylogenetic analysis of cat gene clusters. The phylogenetic tree was generated using MEGA 5.0 with maximum parsimony and 1000 bootstrap replicates. Reference DNA sequences were selected by BLAST searches in the NCBI database.

**Fig. 11:**
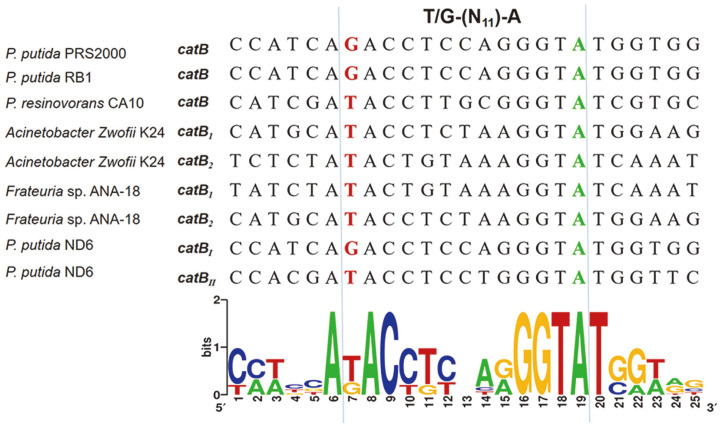
LOGO diagrams of the binding sites occupied by CatR. The sequence was derived from the *catR*-B intergenic region of the reported catRBCA gene clusters, including *P. putida* PRS2000 [13], *P. putida* RB1 [11], *Pseudomonas resinovorans* strain CA10 [40], *Acinetobacter lwoffii* K24 [41], *Frateuria* sp. ANA-18 [19, 23], and *P. putida* ND6. The LOGO diagrams were built using all the predicted binding sites for the respective CatR in all studied genomes in all *catR-B* intergenic regions. The sequence between the two dashed lines is an inverted repeat (T-N_11_-A ) or an incomplete inverted repeat (G-N_11_-A ). The T/G and A residues of the T/G-N_11_-A motif were marked in red and green, respectively.

To analyze the diversity of the two catechol-degrading gene clusters in the ND6 genome, 24 sequences of different cat gene clusters were downloaded from NCBI to construct a phylogenetic tree (Fig. 10). Overall, the phylogenetic analysis showed that the cat gene clusters have significant phylogenetic diversity, and could be divided into two main groups depending on the genus. Most *Pseudomonas* spp. have two *cat* clusters, which constituted one group, including *cat_I_* and *cat_II_* of the ND6 strain. In the *Pseudomonas* spp. group, most second copies of the cat gene clusters, including *catR_II_B_II_C_II_A_II_* of *P. putida* ND6, formed a monophyletic clade. By contrast, most *cat_1_* gene clusters, including *catR_I_B_I_C_I_A_I_* of *P. putida* ND6, formed a separate clade, indicating that *cat_I_* and *cat_II_* of ND6 have different evolutionary origins. Interestingly, in three *Burkholderia* sp. strains (GenBank: CP001052.1, GenBank: AB035483.1 and GenBank: CP026112.1), *catA* was found to be transcribed individually while *catR*-*catB*-*catC* was transcribed in the opposite direction, which is different from the common order *catB*-*catC*/*catA*-*catA*/*catC*. Therefore, the expression of C12O, encoded by *catA*, must be very adaptable to respond to the environment. However, gene clusters with different transcription sequences have a far-reaching relationship. Consistent with a previous study[38], analysis of an alignment of nine intergenic regions of *catB* downloaded from NCBI confirmed the presence of two types of binding motifs, with conserved sequences T-N_11_-A and mutant sequences G-N_11_-A (Fig. 11). In agreement with a report by Parsek *et al*. [37], the mutation of a T to a G resulted in an increase in the binding of CatR_I_ to both the *catB_I_* and *catB_II_* promoters (Fig. 9).

## Discussion

A number of aerobic biodegradation pathways of aromatic compounds like phenol, benzoate, or naphthalene converge into catechol ring cleavage [42]. Our previous study showed that each C12O isozyme in *P. putida* ND6, encoded by the separate *catA* genes on the chromosome and the large pND6-1 plasmid, belonged to independent branches of the phylogenetic tree and have different enzymatic properties [17]. The two *catA* genes located on the chromosome (Fig. 1) were found to significantly contribute to the fitness of the host strain that is adapted to high concentrations of naphthalene [27]. The phenotypic determination showed that the growth curve of *P. putida* ND6-Δ*catAnahH* decreased slightly compared to the wild type, indicating that the two catechol *ortho*-cleavage clusters in the genome play an important role but are not the only genes with this function. In nature, many bacteria grow and develop by catechol *ortho*- and *meta*-cleavage pathways to adapt to the presence of aromatics in the environment [3, 43]. The results of RT-qPCR and the catechol dioxygenase enzyme activity assay supported this idea. In addition to *P. putida* ND6, many other strains, especially of *Pseudomonas* and *Burkholderia* spp., possess multiple cat gene clusters. In *Burkholderia* sp. strain TH2, although *catA_2_* is not indispensable for the growth on 2CB, the presence of the *cat_2_* gene cluster is beneficial [44]. Similar observations were made in *Acinetobacter lwoffii* K24, and *Frateuria* species ANA-18 [19]. Thus, the presence of multiple *cat* genes or clusters in the same cell is a widespread phenomenon[14] that may help the host adapt to changing environments by either reducing the intracellular concentration of catechol, which is a toxic intermediate of the degradation of various aromatic compounds, or by substituting for each other to circumvent harmful mutations [3].

It is interesting to note the different arrangement of *cat* genes in Fig. 10. Most of them are arranged in the order *catB*-*catC*/*catA*-*catA*/*catC*, while the catR gene is divergently transcribed from the operon. Furthermore, the *catA* gene is transcribed separately from *catR*-*catB*-*catC* as arranged in *Paraburkholderia terrae* strain DSM 17804, *Burkholderia* sp. strain TH2 and *Paraburkholderia phytofirmans* strain PsJN. At the same time, we found that *catA* is under independent transcriptional regulation in the ND6 strain, while the *catBCA* operon is co-transcribed. Consequently, we assumed that individual transcription of the *catA* gene might be important and not an accidental evolutionary event. Notably, *catA* encodes the rate-limited enzyme C12O in the catechol *ortho*-cleavage pathway, so the abundance of C12O should be regulated sensitively and quickly to adapt to the concentration of the substrate. Therefore, the independent transcription of *catA* might represent a positively selected genetic adaptation [3].

The intricate regulatory interplay of multiple cat gene clusters suggests an intimate mutual adaptation [45]. For instance, CatR is able to activate the *clcABD* promoter but ClcR cannot activate the *catBCA* promoter in *P. putida* PRS2000 [46]. As a result, transcription from the clc promoter is repressed by the TCA-cycle intermediates succinate, citrate and fumarate, while the presence of these organic acids does not affect the transcription from the cat promoter. This difference provides some flexibility to respond to different environmental signals in addition to the presence of the inducer [47]. Similarly, the question of how the two *catBCA* gene clusters are regulated in *P. putida* ND6 was investigated. We found that the core transcriptional activation mechanisms of the two *ortho*-cleavage operons are conserved. First, both can be induced by CCM. Second, CatR_I_ and CatR_II_ are the native regulatory proteins of the *cat_I_* cluster and *cat_II_* cluster, respectively. Third, in the absence of CCM, CatR_I_ and CatR_II_ bind to the RBS, which contains a T/G-N_11_-A motif, presumably allowing CatR to negatively regulate its own expression, while in the presence of CCM, CatR_I_ and CatR_II_ occupy an adjacent downstream site, designated as the ABS. Based on these conserved characters, CatR_I_ and CatR_II_ share functional similarities which allow them to complement each other’s mutants at the transcriptional level. Different from CatR and ClcR of *P. putida*, CatR_I_ and CatR_II_ can activate the promoters of the other, which provides another level of flexibility to respond to harmful mutations of each regulator. Additionally, EMSA demonstrated that CatR_I_ binds to both the *cat_I_* and *cat_II_* promoters with high affinity, while CatR_II_ binds weakly. These observations were confirmed by *lacZ* transcriptional-fusion expression experiments, which indicated that CatR_I_ might competitively bind the promoter region of *cat_II_* and initiate transcription more effectively (Table 3). A mutation in the binding motif from T-N_11_-A (*cat_II_*) to G-N_11_-A (*cat_I_*) may explain the difference of binding characteristics. In conclusion, the two cat gene clusters in *P. putida* ND6 can cross-regulate each other as a result of similar evolutionary origins, but they diverged due to the accumulation of mutations that appear to be evolutionarily advantageous.

In this study, we found that the *catC_I_*/*catC_II_* and *catA_I_*/*catA_II_* genes are transcribed independently, in addition to the co-transcription of the *catBCA* operon. We are conducting further experiments to demonstrate why and how the regulation of *cat* genes in *P. putida* ND6 is so complex.

## Figures and Tables

**Table 1:** Strains and plasmids used in this study.

Strains/plasmids		Genotype and description	Source/reference
*E. coli*	JM109	cloning strain	Novagen
	S17-1	*recA pro hsdR* RP4-2-Tc::Mu-Km::Tn7; donor strain for conjugation;	[28]
	BL21	Expression strain	Novagen
*P. putida*	ND6	Cb^r^	[16]
	ND6-∆*catR_I_*	*catR_I_* mutant of ND6；Cb^r^, Kan^r^	This study
	ND6-∆*catR_II_*	*catR_II_* mutant of ND6；Cb^r^, Kan^r^	This study
	ND6-∆*catA*	*catA* mutant of ND6；Cb^r^, Kan^r^	This study
	ND6-∆*catAnahH*	*catA* and *nahH* mutant of ND6；Cb^r^, Kan^r^, Cm^r^	This study
	NC	ND6 containing plasmid pDN19*lac*Ω; Cb^r^, Sp^r^, Sm^r^	This study
	NP1	ND6 containing plasmid pDB1; Cb^r^, Sp^r^, Sm^r^	This study
	NP2	ND6 containing plasmid pDB2; Cb^r^, Sp^r^, Sm^r^	This study
	ND1P1	ND6-∆*catR_I_* containing plasmid pDB1; Cb^r^, Kan^r^, Sp^r^, Sm^r^	This study
	ND1P2	ND6-∆*catR_I_* containing plasmid pDB2; Cb^r^, Kan^r^, Sp^r^, Sm^r^	This study
	ND2P1	ND6-∆*catR_II_* containing plasmid pDB1; Cb^r^, Kan^r^, Sp^r^, Sm^r^	This study
	ND2P2	ND6-∆*catR_II_* containing plasmid pDB2; Cb^r^, Kan^r^, Sp^r^, Sm^r^	This study
Plasmid	pUC18-T simple	Cloning vector; Amp^r^	TransGen
	pEX18Tc	Gene replacement; Tc^r^, *oriT*^+^ *sacB*^+^	[29]
	pDN19lacΩ	Broad host range shuttle vector; Sp^r^, Sm^r^	[30]
	pEASY-Blunt	Source of *kan* gene; Amp^r^, Kan^r^	TransGen
	pXMJ19	Source of *cat* gene; Amp^r^, Cm^r^	TransGen
	pEX18Tc-R_I_Km	pEX18Tc with ∆*catR_I_*::Kan^r^; Tet^r^, Kan^r^	This study
	pEX18Tc-R_II_Km	pEX18Tc with ∆*catR_II_*::Kan^r^; Tet^r^, Kan^r^	This study
	pEX18Tc-AKm	pEX18Tc with ∆*catA*::Kan^r^; Tet^r^, Kan^r^	This study
	pEX18Tc-HCm	pEX18Tc with ∆*nahH*::Cm^r^; Tet^r^, Cm^r^	This study
	pEX5-catR_I_	CatR_I_ protein expression vector; Kan^r^	This study
	pEX5-catR_II_	CatR_II_ protein expression vector; Kan^r^	This study
	pUC19c-*catB_I_*	pUC19c with *catB_I_* promoter; Ap^r^	This study
	pUC19c-*catB_II_*	pUC19c with *catB_II_* promoter; Ap^r^	This study
	pDB_I_	*cat_I_* promoter inserted between the *Eco*RI and *Bam*HI sites of pDN19lacΩ; Sp^r^, Sm^r^	This study
	pDB_II_	*cat_II_* promoter inserted between the *Eco*RI and *Bam*HI sites of pDN19lacΩ; Sp^r^, Sm^r^	This study

*Cb^r^, carbenicillin resistant; Kan^r^, kanamycin resistant; Cm^r^, Chloramphenicol resistant; Sp^r^, spectinomycin resistant; Sm^r^, streptomycin resistant; Amp^r^, ampicillin resistant; Tet^r^, tetracycline resistant; Gm^r^, gentamicin resistant.

**Table 2:** Primers used in this study.

Primer name	Sequence (5’–3’)	Description
R_I_L-F	GTGATGTGGCCGAATGCCTC	Used in the creation of ND6-∆*catR_I_* mutant
R_I_L-R	CGGCATGAGCATTCGTGTC	Used in the creation of ND6-∆*catR_I_* mutant
R_I_R-F	GGCCGAAGCCAATGTCGA	Used in the creation of ND6-∆*catR_I_* mutant
R_I_R-R	GCTTTCGATGCCGGACTTG	Used in the creation of ND6-∆*catR_I_* mutant
R_II_L-F	GACGGCCAAGTCGATGGTG	Used in the creation of ND6-∆*catR_II_* mutant
R_II_L-R	TAGGCCAGCCGATATCGCT	Used in the creation of ND6-∆*catR_II_* mutant
R_II_R-F	CCTGATAGATAGCCGCGTCG	Used in the creation of ND6-∆*catR_II_* mutant
R_II_R-R	AGCAAGCGAAGAATGACCAAGG	Used in the creation of ND6-∆*catR_II_* mutant
AL-F	GGGCCGCTTGATCAACGTCGT	Used in the creation of ND6-∆*AH* mutant
AL-R	ATCTCAGGCAGGTTGGAAATAG	Used in the creation of ND6-∆*AH* mutant
AR-F	CAGTCCGAATACAACCTGCGC	Used in the creation of ND6-∆*AH* mutant
AR-R	CCGAAGGCGACAAAGCTGGAG	Used in the creation of ND6-∆*AH* mutant
HL-F	GCGCCGCTGGACGTGACAATGC	Used in the creation of ND6-∆*AH* mutant
HL-R	TCCAGTACACGCAGTTGCACG	Used in the creation of ND6-∆*AH* mutant
HR-F	TTATTTCTTCGACCCGTCCGG	Used in the creation of ND6-∆*AH* mutant
HR-R	GGCCGGTCCGACTTTCCAGGT	Used in the creation of ND6-∆*AH* mutant
kan-F	GGGCGGTTTTATGGACAGC	Used in the creation of ND6-∆*catR_I_*, ND6-∆*catR_II_*, ND6-∆AH mutant
kan-R	CGGTGCTCAACGGGAATC	Used in the creation of ND6-∆*catR_I_*, ND6-∆*catR_II_*, ND6-∆*AH* mutant
cat-F	CTTAAAAAAATTACGCCCCGC	Used in the creation of ND6-∆*AH* mutant
cat-R	TGATCGGCACGTAAGAGGTTC	Used in the creation of ND6-∆*AH* mutant
catB_I_P-F	AGCACGGTGCAGGTCTGTTC	Used for construction *catB_I_*-*lacZ* fusions
catB_I_P-R	GCCCGAGTGATGCGTTTAC	Used for construction *catB_I_*-*lacZ* fusions
catB_II_P-F	CAAGCTGTTTGAGCAAGGTGTC	Used for construction *catB_II_*-*lacZ* fusions
catB_II_P-R	TAAGGCATCAATACCCATCG	Used for construction *catB_II_*-*lacZ* fusions
CatR_I_-F	ACTTGATTGTTGAAGGATT	Used for CatR_I_ protein expression
CatR_I_-R	TCAGAGTGCCTGTTGCTC	Used for CatR_I_ protein expression
CatR_II_-F	GTGGAGCTCAGGCAT	Used for CatR_II_ protein expression
CatR_II_-R	TTATCGATTGATGGTCG	Used for CatR_II_ protein expression
q16S-F	CGGATCGCAGTCTGCAACTC	16s gene for RT-qPCR
q16S-R	ACACCGTGGTAACCGTCCTCC	16s gene for RT-qPCR
qcatA_I_-F	AGGAATGCCTGGACCTGCTCG	*catA_I_* gene amplification for RT-qPCR
qcatA_I_-R	CGAATGACAACTCGGCAAAGCG	*catA_I_* gene amplificationfor RT-qPCR
qcatB_I_-F	CTGGCATTGCCTTGTACGG	*catB_I_* gene amplification for RT-qPCR
qcatB_I_-R	AGGCGCTGTTCGTCCAATG	*catB_I_* gene amplification for RT-qPCR
qcatC_I_-F	ACATGGACCCGGCCAAGG	*catC_I_* gene amplification for RT-qPCR
qcatC_I_-R	TCAGCGATCGTCGCTGTG	*catC_I_* gene amplification for RT-qPCR
qcatA_II_-F	AAGGGCTAATCGGCGAAGTG	*catA_II_* gene amplification for RT-qPCR
qcatA_II_-R	GCTGTTCGGCAGTCTCAACC	*catA_II_* gene amplification for RT-qPCR
qcatB_II_-F	GGTGCGGCTCAATCAACG	*catB_II_* gene amplification for RT-qPCR
qcatB_II_-R	GCCTGGGCTATCTGGGCAG	*catB_II_* gene amplification for RT-qPCR
qcatC_II_-F	CAAGTGGCTGCCCGCTTG	*catC_II_* gene amplification for RT-qPCR
qcatC_II_-R	AGCGCTTCGACACTGTCCACA	*catC_II_* gene amplification for RT-qPCR
catB_I_C_I_-F	GGGCCTGACATTGGACGAAC	The region of *catB_I_* gene and *catC_I_* gene; RT-PCR
catB_I_C_I_-R	ACACAGGCCGTCGACCTCA	The region of *catB_I_* gene and *catC_I_* gene; RT-PCR
catC_I_A_I_-F	CGTACATGGACATTGAGGTCGA	The region of *catC_I_* gene and *catA_I_* gene; RT-PCR
catC_I_A_I_-R	AGGTCGAGGAAGTGCTCGATG	The region of *catC_I_* gene and *catA_I_* gene; RT-PCR
catB_II_C_II_-F	GATCAGCTGCAATGGCACAC	The region of *catB_II_* gene and *catC_2_* gene; RT-PCR
catB_II_C_II_-R	AGCGCTTCGACACTGTCC	The region of *catB_II_* gene and *catC_II_* gene; RT-PCR
catC_II_A_II_-F	CAAGTGGCTGCCCGCTT	The region of *catC_II_* gene and *catA_II_* gene; RT-PCR
catC_II_A_II_-R	CGATAAACAACGTGGTGGCAAC	The region of *catC_II_* gene and *catA_II_* gene; RT-PCR
M13F-11	CGCCAGGGTTTTCCCAGTCACGAC	TaqMan primer used for *catB_I_* or *catB_II_* EMSA and DNase I footprinting template
M13R-12	AGCGGATAACAATTTCACACAGGA	TaqMan primer used for *catB_I_* or *catB_II_* EMSA and DNase I footprinting template
catB_I_-pe	TGTGTTCAATCAGCGCGCTTGTC	*CatB_I_* gene-specific primer, Primer extension
catB_II_-pe	GCTGCTGTCATTTCAGGTTCCATC	*CatB_II_* gene-specific primer, Primer extension

**Table 3:** Determination of relative promoter activities via β-galactosidase expression (measured in Miller units).

Strains	NC	NP1	NP2	ND1P1	ND1P2	ND2P1	ND2P2
MMB + glucose	8.37	151.30	15.89	8.09	9.45	196.37	8.19
MMB + naphthalene	8.30	199.31	11.52	11.87	9.94	297.38	11.54

*The β-ga*lac*tosidase activity was determined in the following strains: NC (ND6 containing pDN19*lac*Ω); NP1 (ND6 containing pDN19*lac*Ω + *cat_I_* promoter); NP2 (ND6 containing pDN19*lac*Ω + *cat_II_* promoter); ND1P1 (ND6-Δ*catR_I_* containing pDN19*lac*Ω + *cat_I_* promoter); ND1P2 (ND6-Δ*catR_I_* containing pDN19*lac*Ω + *cat_II_* promoter); ND2P1 (ND6-Δ*catR_II_* containing pDN19*lac*Ω + *cat_I_* promoter); ND2P2 (ND6-Δ*catR_II_* containing pDN19*lac*Ω + *cat_II_* promoter).

## References

[1] Linger JG, Vardon DR, Guarnieri MT, Karp EM, Hunsinger GB, Franden MA (2014). Lignin valorization through integrated biological funneling and chemical catalysis. Proc. Natl. Acad. Sci. USA.

[2] Mallick S (2019). Biodegradation of acenaphthene by *Sphingobacterium* sp. strain RTSB involving trans-3-carboxy-2-hydroxybenzylidenepyruvic acid as a metabolite. Chemosphere.

[3] Setlhare B, Kumar A, Mokoena MP, Olaniran AO (2018). Catechol 1,2-Dioxygenase is an analogue of Homogentisate 1,2-Dioxygenase in *Pseudomonas chlororaphis* Strain UFB2. Int. J. Mol. Sci..

[4] Singh SN, Kumari B, Upadhyay SK, Mishra S, Kumar D (2013). Bacterial degradation of pyrene in minimal salt medium mediated by catechol dioxygenases: enzyme purification and molecular size determination. Bioresour. Technol..

[5] Wackett LP (2003). *Pseudomonas putida* a versatile biocatalyst. Nat. Biotechnol..

[6] Jimenez JI, Perez-Pantoja D, Chavarria M, Diaz E, de Lorenzo V (2014). A second chromosomal copy of the *catA* gene endows *Pseudomonas putida* mt-2 with an enzymatic safety valve for excess of catechol. Environ. Microbiol..

[7] Caposio P, Pessione E, Giuffrida G, Conti A, Landolfo S, Giunta C (2002). Cloning and characterization of two catechol 1,2-dioxygenase genes from *Acinetobacter radioresistens* S13. Res. Microbiol..

[8] Harwood CS, Parales RE (1996). The beta-ketoadipate pathway and the biology of self-identity.. Annu. Rev. Microbiol..

[9] Tumen-Velasquez MP, Laniohan NS, Momany C, Neidle EL (2019). Engineering CatM, a LysR-type transcriptional regulator, to respond synergistically to two effectors. Genes (Basel).

[10] Parsek MR, Shinabarger DL, Rothmel RK, Chakrabarty AM (1992). Roles of CatR and *cis*,*cis*-muconate in activation of the *catBC* operon, which is involved in benzoate degradation in *Pseudomonas putida*. J. Bacteriol..

[11] Chugani SA PM, Hershberger CD, Murakami K, Ishihama A, Chakrabarty AM (1997). Activation of the *catBCA* promoter: probing the interaction of CatR and RNA polymerase through in vitro transcription. J. Bacteriol..

[12] Tover A, Ojangu EL, Kivisaar M (2001). Growth medium composition-determined regulatory mechanisms are superimposed on CatR-mediated transcription from the *pheBA* and *catBCA* promoters in *Pseudomonas putida*. Microbiology.

[13] Rothmel RK, Aldrich TL, Houghton JE, Coco WM, Ornston LN, Chakrabarty AM (1990). Nucleotide sequencing and characterization of *Pseudomonas putida* catR: a positive regulator of the *catBC* operon is a member of the LysR family. J. Bacteriol..

[14] Kohlstedt M, Starck S, Barton N, Stolzenberger J, Selzer M, Mehlmann K (2018). From lignin to nylon: cascaded chemical and biochemical conversion using metabolically engineered *Pseudomonas putida*. Metab. Eng..

[15] Pi H, Helmann JD (2018). Genome-wide characterization of the fur regulatory network reveals a link between catechol degradation and bacillibactin metabolism in *Bacillus subtilis*. mBio.

[16] Li S, Zhao H, Li Y, Niu S, Cai B (2012). Complete genome sequence of the naphthalene-degrading *Pseudomonas putida* strain ND6. J. Bacteriol..

[17] Li S, Qin K, Li H, Guo J, Li D, Liu F (2018). Cloning and characterisation of four *catA* genes located on the chromosome and large plasmid of *Pseudomonas putida* ND6. Electronic J. Biotechnol..

[18] Kim SI, Leem SH, Choi JS, Chung YH, Kim S, Park YM (1997). Cloning and characterization of two *catA* genes in *Acinetobacter lwoffii* K24. J. Bacteriol..

[19] Murakami S, Takashima A, Takemoto J, Takenaka S, Shinke R, Aoki K (1999). Cloning and sequence analysis of two catecholdegrading gene clusters from the aniline-assimilating bacterium *Frateuria species* ANA-18. Gene.

[20] Suzuki K, Ichimura A, Ogawa N, Hasebe A, Miyashita K (2002). Differential expression of two catechol 1,2-dioxygenases in *Burkholderia* sp. strain TH2. J. Bacteriol..

[21] Tian M, Du D, Zhou W, Zeng X, Cheng G (2017). Phenol degradation and genotypic analysis of dioxygenase genes in bacteria isolated from sediments. Braz. J. Microbiol..

[22] Yoon YH, Yun SH, Park SH, Seol SY, Leem SH, Kim SI (2007). Characterization of a new catechol branch of the beta-ketoadipate pathway induced for benzoate degradation in *Acinetobacter lwoffii* K24. Biochem. Biophys. Res. Commun..

[23] Takashima A, Murakami S, Takenaka S, Aoki K (2001). Regulation by two CatR proteins that differ in binding affinity to *catB* promoters expressing two cat gene clusters. Biosci. Biotechnol. Biochem..

[24] Ahn IS, Ghiorse WC, Lion LW, Shuler ML (1998). Growth kinetics of *Pseudomonas putida* G7 on naphthalene and occurrence of naphthalene toxicity during nutrient deprivation. Biotechnol. Bioeng..

[25] Sota M, Yano H, Ono A, Miyazaki R, Ishii H, Genka H (2006). Genomic and functional analysis of the IncP-9 naphthalenecatabolic plasmid NAH7 and its transposon Tn4655 suggests catabolic gene spread by a tyrosine recombinase. J. Bacteriol..

[26] Dennis JJ, Zylstra GJ (2004). Complete sequence and genetic organization of pDTG1, the 83 kilobase naphthalene degradation plasmid from *Pseudomonas putida* strain NCIB 9816-4. J. Mol. Biol..

[27] Li SS, Hu X, Zhao H, Li YX, Zhang L, Gong LJ (2015). Quantitative analysis of cellular proteome alterations of *Pseudomonas putida* to naphthalene-induced stress. Biotechnol. Lett..

[28] Park W, Jeon CO, Hohnstock-Ashe AM, Winans SC, Zylstra GJ, Madsen EL (2003). Identification and characterization of the conjugal transfer region of the pCg1 plasmid from naphthalene-degrading *Pseudomonas putida* Cg1. Appl. Environ. Microbiol..

[29] Hoang TT, Karkhoff-Schweizer RR, Kutchma AJ, Schweizer HP (1998). A broad-host-range Flp-FRT recombination system for sitespecific ex*cis*ion of chromosomally-located DNA sequences: application for isolation of unmarked *Pseudomonas aeruginosa* mutants. Gene.

[30] Totten PA, Lory S (1990). Characterization of the type a flagellin gene from *Pseudomonas aeruginosa* PAK. J. Bacteriol..

[31] Green M R, Sambrook J (2012). Molecular Cloning: A Laboratory Manual.

[32] Li S, Li X, Zhao H, Cai B (2011). Physiological role of the novel salicylaldehyde dehydrogenase *NahV* in mineralization of naphthalene by *Pseudomonas putida* ND6. Microbiol. Res..

[33] Paulin MM, Novinscak A, St-Arnaud M, Goyer C, DeCoste NJ, Prive JP (2009). Transcriptional activity of antifungal metaboliteencoding genes *phlD* and *hcnBC* in *Pseudomonas* spp. using RT-qPCR. FEMS Microbiol. Ecol..

[34] Fekete RA, Miller MJ, Chattoraj DK (2003). Fluorescently labeled oligonucleotide extension: a rapid and quantitative protocol for primer extension. Biotechniques.

[35] Wang Y, Cen XF, Zhao GP, Wang J (2012). Characterization of a new GlnR binding box in the promoter of *amtB* in *Streptomyces coelicolor* inferred a PhoP/GlnR competitive binding mechanism for transcriptional regulation of *amtB*. J. Bacteriol..

[36] Chugani SA PM, Chakrabarty AM (1998). Transcriptional repression mediated by LysR-type regulator CatR bound at multiple binding sites. J. Bacteriol..

[37] Parsek MR, Ye RW, Pun P, Chakrabarty AM (1994). Critical nucleotides in the interaction of a LysR-type regulator with its target promoter region *catBC* promoter activation by CatR. J. Biol. Chem..

[38] Goethals K, Van Montagu M, Holsters M (1992). Conserved motifs in a divergent nod box of *Arhizobium caulinodans* ORS571 reveal a common structure in promoters regulated by LysR-type proteins. Proc. Natl. Acad. Sci. USA.

[39] Browning DF, Busby SJ (2016). Local and global regulation of transcription initiation in bacteria. Nat. Rev. Microbiol..

[40] Nojiri H, Maeda K, Sekiguchi H, Urata M, Shintani M, Yoshida T (2002). Organization and transcriptional characterization of catechol degradation genes involved in carbazole degradation by *Pseudomonas resinovorans* strain CA10. Biosci. Biotechnol. Biochem..

[41] Kim SI, Ha KS, Leem SH (1999). Differential organization and transcription of the *cat2* gene cluster in aniline-assimilating *Acinetobacter lwoffii* K24. J. Biosci. Bioeng..

[42] Vesely M, Knoppova M, Nesvera J, Patek M (2007). Analysis of *catRABC* operon for catechol degradation from phenol-degrading *Rhodococcus erythropolis*. Appl. Microbiol. Biotechnol..

[43] Chettri B, Singh AK (2019). Kinetics of hydrocarbon degradation by a newly isolated heavy metal tolerant bacterium *Novosphingobium panipatense* P5:ABC. Bioresour. Technol..

[44] Zhao H, Chen D, Li Y, Cai B (2005). Overexpression, purification and characterization of a new salicylate hydroxylase from naphthalene-degrading *Pseudomonas* sp. strain ND6. Microbiol. Res..

[45] Suvorova IA, Gelfand MS (2019). Comparative genomic analysis of the regulation of aromatic metabolism in betaproteobacteria. Front. Microbiol..

[46] Parsek MR, McFall SM, Shinabarger DL, Chakrabarty AM (1994). Interaction of two LysR-type regulatory proteins CatR and ClcR with heterologous promoters: functional and evolutionary implications. Proc. Natl. Acad. Sci. USA.

[47] McFall SM, Chugani SA, Chakrabarty AM (1998). Transcriptional activation of the catechol and chlorocatechol operons: variations on a theme. Gene.

